# Village Poor and London Hospitals

**Published:** 1893-12-16

**Authors:** 


### VILLAGE POOR AND LONDON HOSPITALS.

At a meeting of the Bedford Board of Guardians on Saturday a letter was read from Miss Mary Thomas, of Bletsoe, complaining of the conduct of the Board in refusing to bear the funeral expenses in connection with the death in St. George's Hospital, London, of a labourer named Partridge. It appeared that Partridge, after much persuasion, was sent up as a private patient, the Board undertaking to bear the expenses of the journey to and from London. After undergoing intense suffering the man died, and his body was brought home by rail. Miss Thomas, although objecting to the conduct of the Board, now wrote stating that she would pay for the transit of the corpse if the Board would pay £2 10s. for the coffin. The Clerk pointed out that if the application was granted the Board might be surcharged by the auditor. Several Guardians held that the law was being strained, and pointed out that if the poor knew that they were to be buried in some hole in London they would not leave their villages for treatment in the London hospitals. Law or no law, auditor or no auditor, the Board resolved to pay the £2 10s. asked for. Mr. Hipwell drew attention to the exorbitant rates charged by the railways for the conveyance of corpses. They would carry a living person for 4s. 7d., a horse for 15s., but for the same distance they would charge 57s. for a corpse conveyed in a horse-box. Such high rates often acted as a hardship to the poor who wished to get their dead home from the hospitals.

